# Ultrasound-Induced Destruction of Nitric Oxide–Loaded Microbubbles in the Treatment of Thrombus and Ischemia–Reperfusion Injury

**DOI:** 10.3389/fphar.2021.745693

**Published:** 2022-01-04

**Authors:** Zenghui Liang, Huafang Chen, Xuehao Gong, Binbin Shi, Lili Lin, Fangyi Tao, Qilong Wu, Mingling Fang, Hui Li, Cuitao Lu, Helin Xu, Yingzheng Zhao, Bin Chen

**Affiliations:** ^1^ Department of Ultrasonography, The First Affiliated Hospital of Wenzhou Medical University, Wenzhou, China; ^2^ The Office of Drug Clinical Trial Institution, The First Affiliated Hospital of Wenzhou Medical University, Wenzhou, China; ^3^ Department of Ultrasound, First Affiliated Hospital of Shenzhen University, Second People's Hospital of Shenzhen, Shenzhen, China; ^4^ Department of Pharmaceutics, School of Pharmaceutical Sciences, Wenzhou Medical University, Wenzhou, China

**Keywords:** nitric oxide, thrombus, ischemia–reperfusion injury, ultrasound-targeted microbubbles destruction, endothelial nitric oxide synthase

## Abstract

**Objectives:** Early recanalization of large vessels in thromboembolism, such as myocardial infarction and ischemic stroke, is associated with improved clinical outcomes. Nitric oxide (NO), a biological gas signaling molecule, has been proven to protect against ischemia–reperfusion injury (IRI). However, the underlying mechanisms remain to be explored. This study investigated whether NO could mitigate IRI and the role of NO during acoustic cavitation.

**Methods:** *In vivo*, thrombi in the iliac artery of rats were induced by 5% FeCl_3_. NO-loaded microbubbles (NO-MBs) and ultrasound (US) were used to treat thrombi. B-mode and Doppler US and histological analyses were utilized to evaluate the thrombolysis effect in rats with thrombi. Immunohistochemistry, immunofluorescence, and western blotting were conducted to investigate the underlying mechanisms of NO during acoustic cavitation. *In vitro*, hypoxia was used to stimulate cells, and NO-MBs were employed to alleviate oxidative stress and apoptosis.

**Results:** We developed NO-MBs that significantly improve the circulation time of NO *in vivo*, are visible, and effectively release therapeutic gas under US. US-targeted microbubble destruction (UTMD) and NO-loaded UTMD (NO + UTMD) caused a significant decrease in the thrombus area and an increase in the recanalization rates and blood flow velocities compared to the control and US groups. We discovered that UTMD induced NO generation through activation of endothelial NO synthase (eNOS) *in vivo*. More importantly, we also observed significantly increased NO content and eNOS expression in the NO + UTMD group compared to the UTMD group. NO + UTMD can mitigate oxidative stress and apoptosis in the hind limb muscle without influencing blood pressure or liver and kidney functions. *In vitro*, NO-MBs alleviated oxidative stress and apoptosis in cells pretreated with hypoxia.

**Conclusion:** Based on these data, UTMD affects the vascular endothelium by activating eNOS, and NO exerts a protective effect against IRI.

## 1 Introduction

Thromboembolism includes arterial and venous thrombotic conditions. Arterial thrombosis is the major pathophysiologic event initiating acute myocardial infarction and ischemic stroke, which are prevalent worldwide and leading causes of mortality (Wendelboe and Raskob, 2016). To save damaged tissue, vascular recanalization and rapid recovery of blood flow are of crucial importance in the treatment of thromboembolism (Man et al., 2020). Current conventional therapies for thromboembolism include catheter intervention and pharmacological thrombolysis, both of which have enhanced the prognosis of patients. However, both catheter intervention and pharmacological thrombolysis elicited ischemia–reperfusion injury (IRI), which was characterized by occlusion of the blood supply to an organ followed by the subsequent restoration of perfusion and reoxygenation (Yellon and Hausenloy, 2007). IRI results in tissue injury, which decreases the benefits of recanalization and thereby increases the risk of mortality through a wide range of pathological processes that include reactive oxygen species (ROS), microvascular dysfunction, and cell apoptosis (Eltzschig and Eckle, 2011).

Ultrasound (US)-targeted microbubble destruction (UTMD) has shown great potential in the targeted delivery of drugs, gases, and genes to various organs. The therapeutic potential of UTMD has received great attention as microbubbles (MBs) oscillate and finally collapse, thereby increasing the permeability of the microvasculature and the specific transportation of drugs to tissues (Chowdhury et al., 2020). The delivery of therapeutic gases such as hydrogen sulfide and oxygen through UTMD to target tissues have been shown to be effective. For example, the delivery of hydrogen sulfide to the myocardium with hydrogen sulfide MBs combined with US attenuates myocardial IRI and minimizes side effects (Chen et al., 2016). Using US- and oxygen-loaded MBs result in ROS generation and an enhanced sonodynamic effect in hypoxic tumors (McEwan et al., 2015). These findings indicate that exogenous delivery of NO to tissues could be made feasible by UTMD.

Over the decades, there have been numerous efforts made to use therapeutic gases such as hydrogen, hydrogen sulfide, and carbon monoxide to address IRI in animal experiments (Fujimoto et al., 2004; Elrod et al., 2007; Ohsawa et al., 2007). However, there are few promising therapies for attenuating IRI in clinical practice. Nitric oxide (NO), a well-known biological gas signaling molecule, was proposed to protect against IRI in both animal experiments and clinical practice (Duranski et al., 2005; Lang et al., 2007; Gladwin et al., 2011; Neye et al., 2012; Fix et al., 2015). There is substantial evidence indicating that NO is effective against IRI in other models through complex mechanisms, such as regulating vasomotor tone, platelet aggregation and adhesion, inflammatory and immune responses, and apoptosis (Lefer et al., 1993; Schulz et al., 2004; Hendgen-Cotta et al., 2008; Shinbo et al., 2013). Strategies used in the delivery of NO in therapeutic applications include inhaled NO, nitrite, and NO donors (Bice et al., 2016). However, the bioavailability of NO to target tissues from all delivery strategies is not ideal because of its limited availability to damaged tissues. Novel NO donors have advantages, such as decreasing systemic side effects and increasing therapeutic potential, but are still not specific to damaged tissues.

Previous studies have found that conservative treatment of thrombi goes beyond thrombolytic drugs and that US combined with MBs can effectively dissolve thrombi (Wang et al., 2008; Cui et al., 2017). In a previous study in 1996, Porter and other scholars compared US combined with MBs and US combined with urokinase and discovered that they showed the same thrombolytic effect (Porter et al., 1996). Thrombolysis by US combined with MBs may become an attractive and effective auxiliary means for patients who are unable to receive surgery or thrombolytic drugs due to physical problems. Accumulated evidence indicates that the physical effect of acoustic cavitation (MB oscillation and collapse) can promote thrombolysis (Cintas et al., 2004; Datta et al., 2006; Chen et al., 2014). In a recent study, UTMD dissolved microthrombi and increased NO concentrations in the hind limb muscle (Yu et al., 2017). Nitrite, an NO donor, increased microvascular blood flow and decreased local oxidative stress when combined with UTMD (Yu et al., 2020). In this exploratory study, we attempted to investigate the effects of UTMD on the vascular endothelium and the effect of exogenous NO on IRI.

In this study, we describe the characterization of NO-loaded MBs (NO-MBs) and their ability to increase the bioavailability of NO in the hind limb muscle. The feasibility and effectiveness of the NO delivery strategy were investigated in the hind limb model. We hypothesized that acoustic cavitation has bioeffects on the vascular endothelium, causing NO generation by activating endothelial NO synthase (eNOS). NO-loaded UTMD (NO + UTMD) mitigated IRI caused by vascular recanalization.

## 2 Materials and Methods

### 2.1 Preparation and Characterization of MBs and NO-MBs

MBs were prepared by the lyophilization method. 1,2-diacyl-sn-glycero-3-phosphocholine (Advanced Vehicle Technology Pharmaceutical Ltd, Shanghai, China), 1,2-dioctadecanoyl-sn-glycero-3-phosphocholine (Advanced Vehicle Technology Pharmaceutical Ltd, Shanghai, China), Tween-80 (Solarbio, Beijing, China), and poloxamer 188 (Sigma-Aldrich, MO, United States) were dissolved in tert-butanol and stored at 4°C overnight. The coagulated solution was lyophilized at 5 × 10^−4^ Pa pressure for 24 h (primary drying at −48°C for 20 h and the temperature was gradually raised to 5°C for 4 h). A certain amount of freeze-dried powder was placed in a vial under vacuum. NO was deoxygenated with saturated NaOH solution. About 10 ml of sulfur hexafluoride (SF_6_) or a gaseous mixture of NO and SF_6_ (volume ratio, 1:9) was injected into the vials using a 10-ml syringe to obtain MBs and NO-MBs. The MBs and NO-MBs samples were assessed under an optical microscope (Nikon, Tokyo, Japan). The mean diameter and concentration of the MBs and NO-MBs were measured using a Multisizer 4e Coulter counter (Beckman Coulter Inc., Brea, CA, United States).

### 2.2 Ultrasound-Triggered NO Release From NO-MBs *In Vitro*

The release of NO from NO-MBs was measured by dialysis under sink conditions. The NO-MB solution (1 ml, 1.19 × 10^8^ MBs/ml) was placed in a dialysis bag (MWCO: 3500, Solarbio, Beijing, China) and dialyzed against 100 ml of PBS (receiving buffer). A diagnosis US transducer with a frequency of 1.6 MHz and a mechanical index of 0.7 (S5-1, Philips Medical Systems, MA, United States) was placed into the receiving buffer to destroy the NO-MBs. The release of NO from NO-MBs was measured at 5, 10, 20, 30, 40, 60, 80, 100, and 120 min. The measurement of the release of NO from NO-MBs to the receiving buffer was based on the formation of nitrite from the reaction of NO with oxygen (4NO + O_2_ + 2H_2_O → 4NO_2_^−^+ 4H^+^). The concentration of nitrite in the receiving solution was measured with a NO commercial assay kit (Solarbio, Beijing, China).

### 2.3 Specific Delivery of NO by UTMD in Hind Limb Muscle

The NO content was measured in the hind limb muscle and kidneys of healthy non-thromboembolism rats. The rats were randomly divided into three groups with three rats in each group: 1) control group—normal saline was injected at the speed of 5 ml/h into the right internal jugular vein for 15 min; 2) NO-MBs group—NO-MB suspension (1.19 × 10^8^ MBs/ml) was injected at the speed of 5 ml/h into the right internal jugular vein for 15 min without US irradiation; and 3) NO + UTMD group—NO-MB suspension (1.19 × 10^8^ MBs/ml) was injected into the right internal jugular vein at the speed of 5 ml/h, and the rats received US irradiation for 15 min. After treatment, the hind limb muscle and kidneys were obtained, homogenized, and centrifuged for 10 min (12,000 rpm, 4°C). The NO content of the hind limb muscle and kidney tissue was measured by a NO commercial assay kit (Solarbio, Beijing, China).

### 2.4 Contrast-Enhanced Ultrasound Imaging in Rat Hind Limb

Contrast-enhanced US (CEUS) imaging was performed in healthy rats without thrombosis. The hair of the rat's left hind limb was removed. The transducer was positioned vertically above the femoris muscle. CEUS was performed with the US system (Philips Medical Systems, MA, United States) and imaging transducer (L12-3). The depth and gain settings were optimized from the beginning and kept unchanged throughout the experiment. MBs (1.10 × 10^8^ MBs/ml, 5 ml/h) or NO-MBs (1.19 × 10^8^ MBs/ml, 5 ml/h) were injected into the jugular veins of the rats. Then, a diagnostic US (S5-1) transducer with a frequency of 1.6 MHz and a mechanical index of 0.7 was utilized for 15 min to destroy MBs or NO-MBs *in vivo*. In addition, a bolus injection of 0.5 ml of MB or NO-MB suspension was given *via* the lateral tail vein. Immediately after the bolus injection, CEUS images were collected to show the circulation time of MBs and NO-MBs in the hind limb. CEUS was performed after MB or NO-MB infusion until the MBs were fully cleared. The US signal intensities in the hind limb were quantified by the QLab software.

### 2.5 *In Vitro* Cell Experiments

#### 2.5.1 Cell Culture and Treatment

Human umbilical vein endothelial cells (HUVECs) and rat skeletal muscle cells (L6) were cultured in Dulbecco's modified Eagle's medium supplemented with 10% fetal bovine serum and 100 U/ml penicillin–streptomycin and maintained at 37°C in humidified conditions with 5% CO_2_. Cells that reached 60–80% confluency from passages 3 to 6 were used in the experiments. To simulate IRI events, cells were seeded in plates with hypoxia (3% O_2_) for 2 h at room temperature. Then, the cells were subjected to a normoxic (20% O_2_) environment, and the supernatant culture medium was replaced with culture medium containing MBs or NO-MBs for 12 h.

#### 2.5.2 Cell Viability in the Presence of MBs and NO-MBs

The viability of L6 cells and HUVECs was evaluated by the MTT assay. The cells were seeded into a 96-well culture plate at 6 × 10^3^ cells/well. The cells were treated with solutions with different concentrations of MBs (10^7^, 10^6^, and 10^5^ MBs/ml) and NO-MBs (10^7^, 10^6^, and 10^5^ MBs/ml) for 12 h. In addition, after hypoxia for 2 h, the cell culture medium was replaced with fresh medium containing different concentrations of MBs (10^6^ and 10^5^ MBs/ml) or NO-MBs (10^6^ and 10^5^ MBs/ml) for 12 h. After different treatments, the viability of cells was measured by the MTT assay.

#### 2.5.3 The Protective Effect of NO-MBs on HUVECs and L6 Cells

To assess the antioxidant effect of NO-MBs, HUVECs and L6 cells were pretreated with hypoxia for 2 h and then incubated with MBs (10^6^ MBs/ml) or NO-MBs (10^6^ MBs/ml) for 12 h. The production of ROS in HUVECs and L6 cells was determined by the DCFH-DA (Beyotime, Shanghai, China) staining method and observed with a fluorescence microscope (Olympus Corp., Tokyo, Japan). In addition, after treatment for 12 h, the cells were harvested, homogenized with PBS, and centrifuged for 10 min (8,000 ×*g*, 4°C). Superoxide dismutase (SOD) and malondialdehyde (MDA) were measured using a commercial assay kit according to the manufacturer's instructions (Solarbio, Beijing, China).

Flow cytometry, Hoechst 33258, and TUNEL staining were performed to determine the antiapoptotic effect of NO-MBs. Briefly, HUVECs and L6 cells were pretreated with hypoxia for 2 h, and then MB and NO-MB solutions were added to the petri dish and reoxygenated at room temperature for 12 h. The cells were stained with Hoechst 33258 for 10 min to observe the changes in nuclear morphology through a fluorescence microscope (Olympus Corp., Tokyo, Japan). To quantitatively examine the number of apoptotic cells, cells were fixed in 4% paraformaldehyde and permeabilized with 0.3% PBS–Triton X-100. Apoptotic cells were detected by using a TUNEL kit (Roche, Germany) and observed using a confocal laser microscope (Nikon, Tokyo, Japan). Finally, the cells were harvested, and Annexin V-FITC staining and PI staining solutions were used to stain the cells for 20 min at room temperature. Cell apoptosis was determined by using the Annexin V-FITC apoptosis detection kit (Beyotime, Beijing, China) and then analyzed with a flow cytometer (Beckman Coulter; CytoFLEX). The data were analyzed with FlowJo Win 7.6.1 software.

### 2.6 *In Vivo* Experiments on Left Iliac Arterial Thrombosis

#### 2.6.1 Left Iliac Arterial Thrombosis Model and *In Vivo* Experimental Protocol

**FIGURE 1:**
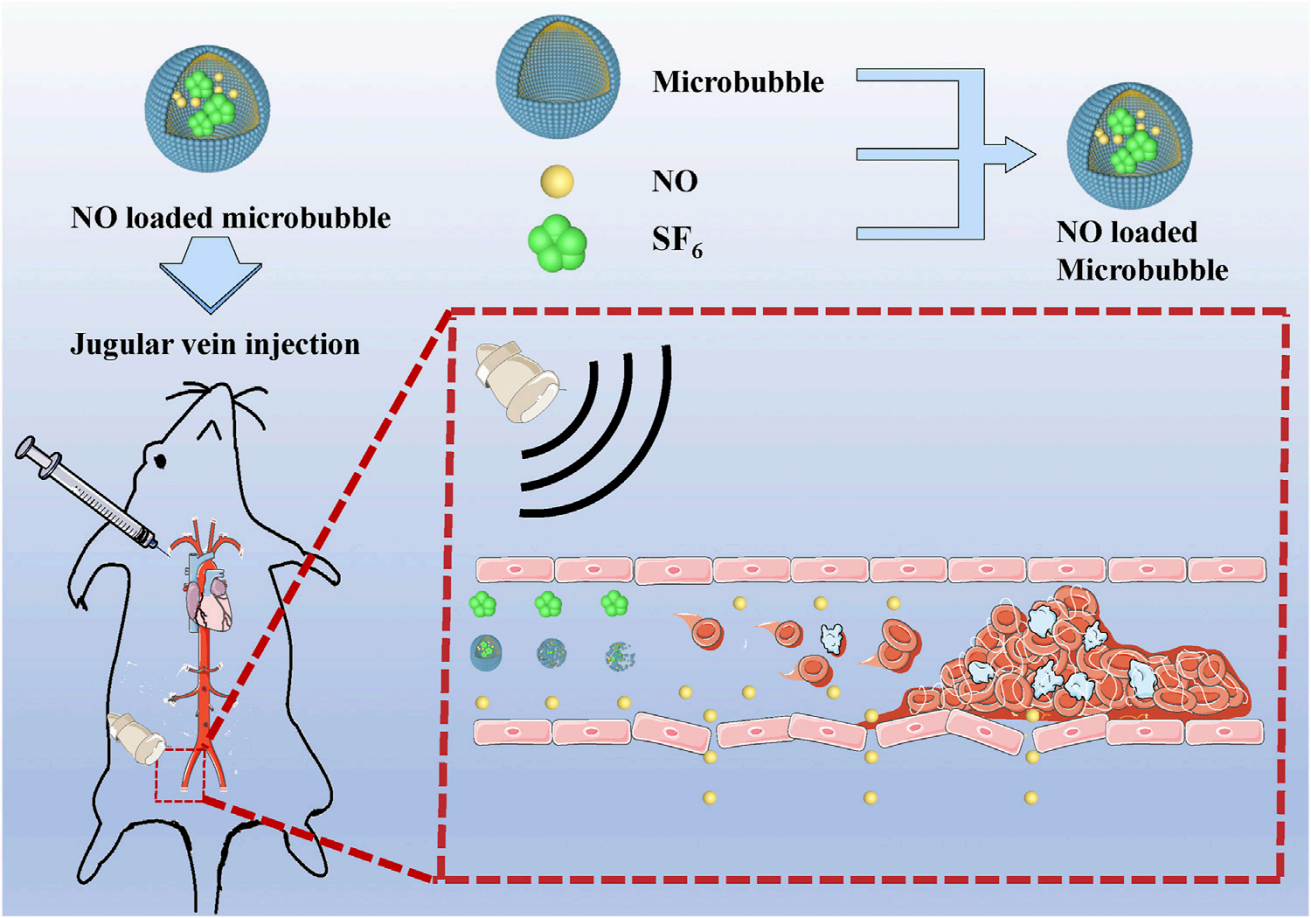
Schematic representation of the treatment process of NO + UTMD *in vivo*.

Adult male Sprague Dawley rats were obtained from the Wenzhou Medical University Animals Breeding Field. On the day of the surgery, the rats were anesthetized, and then a lateral incision was made in their left lower abdomen to expose the artery approximately 0.5 cm below the aortic bifurcation and to isolate it from the surrounding tissue. A small piece of plastic was placed under the artery to avoid damaging the surrounding tissue. A 1-cm-long filter paper containing 5% FeCl_3_ was placed on the surface of the isolated artery for 5 min. After removal of the filter paper, the artery was rinsed with 0.9% sterile saline, and then the incision was stitched closed. The thrombus was observed for 2 h before treatment to guarantee its stability. B-mode and pulsed wave (PW) Doppler US were used to determine whether a completely occluded blood clot had formed and remained stable. If not, 5% FeCl_3_ was applied again for 5 min of thrombosis until successful thrombosis was confirmed. PW Doppler and B-mode US were used to confirm the formation of thrombosis in the left iliac artery. The US probe was placed on the femoris muscle to trigger NO release, as shown in Figure 1. Using a Philips US system, an S5-1 sector array transducer (frequency, 1.6 MHz; MI, 0.7) was utilized for acoustic thrombolysis along the longitudinal direction of the blood vessel. The rats were randomly distributed into four groups with six rats in each group: 1) the control group—normal saline was injected into the right internal jugular vein at a speed of 5 ml/h; 2) the US group—only US irradiation was applied for 15 min; 3) the UTMD group—MB suspension (1.10 × 10^8^ MBs/ml) was injected into the right internal jugular vein at a speed of 5 ml/h, and the rats simultaneously received US irradiation for 15 min; and 4) the NO + UTMD group—NO-MBs suspension (1.19 × 10^8^ MBs/ml) was injected into the right internal jugular vein at a speed of 5 ml/h, and the rats simultaneously received US irradiation for 15 min. After treatment, a blood pressure meter (Softron Biotechnology, Beijing, China) was used to measure the heart rate and blood pressure. The NO content of the hind limb muscle and kidney tissue was measured.

#### 2.6.2 B-Mode Ultrasound Imaging

A Philips US system (Philips Medical Systems, MA, United States) and L12-3 transducer were used to evaluate iliac artery thrombolysis in rats. B-mode and PW Doppler US were used to observe thrombus, the internal diameter, and the blood flow velocity of the iliac artery in the rats. The total exposure time of US imaging for each rat was less than 3 min.

#### 2.6.3 Histological Examinations

To confirm the existence of thrombi *in vivo* and the effect of thrombolysis, iliac artery specimens from the rats were embedded in paraffin and sliced (5 µm). The sliced samples were deparaffinized and stained with hematoxylin and eosin (H&E). Then, immunohistochemical staining was performed on deparaffinized tissue sections with water bath–induced antigen retrieval. Endogenous peroxidase was inactivated by incubating in 3% H_2_O_2_ (containing 80% methanol) for 10 min. Nonspecific binding was blocked by 5% goat serum for 45 min. The primary antibodies, including anti-Caspase-3 antibody (a11593, ABclonal), anti-Caspase-12 antibody (ab62484, Abcam), anti-BAX-antibody (50599-2-Ig, Proteintech), and anti-Bcl-2 antibody (ab7973, Abcam), were added to the sections overnight at 4°C. The sections were washed with PBS and covered with horseradish peroxidase (HRP)–conjugated secondary goat anti-rabbit IgG for 1 h at 37°C. Finally, the cells were stained with diaminobenzidine (DAB) and counterstained with hematoxylin.

#### 2.6.4 Quantification of the Antioxidative Stress Effects of NO-MBs *In Vivo*

The skeletal muscle tissues were embedded vertically in tissue cutting temperature–freezing medium, snap-frozen, cross-sectioned at intervals of 5 μm and incubated with dihydroethidium (DHE; Beyotime, Shanghai, China) at 37°C for 30 min to detect the production of ROS in the tissues. DHE appeared as red fluorescence (excitation wavelength: 535 nm, emission wavelength: 610 nm). All samples were observed with a confocal laser microscope (Nikon, Tokyo, Japan). The fluorescence intensity of ROS in each high-powered field was analyzed by ImageJ 7.6.1. In addition, the skeletal muscle tissues were harvested, homogenized with PBS, and centrifuged for 10 min (8,000 ×*g*, 4°C). SOD and MDA were measured using a commercial assay kit according to the manufacturer's instructions (Solarbio, Beijing, China).

### 2.7 Western Blotting

Skeletal muscle tissues were collected and homogenized in RIPA lysis buffer containing protease inhibitors. The total protein concentrations were determined by the BCA protein assay. The protein samples were separated by 12% sodium dodecyl sulfate–polyacrylamide gel electrophoresis and transferred onto polyvinylidene difluoride membranes for western blot analysis. After blocking with 5% bovine serum albumin for 2 h, the membranes were then incubated with anti-eNOS (1:500 dilution), anti-iNOS (1:200 dilution), anti-Bax (1:1,000 dilution), anti-Bcl-2 (1:200 dilution), and anti-β-actin (1:1,000 dilution) antibodies as primary antibodies at 4°C overnight. The membranes were washed before incubation with HRP-conjugated goat anti-IgG (H + L) secondary antibody (1:10,000 dilution) for 1 h at room temperature. The relative band density was calculated using ImageJ software, and an antibody against *β*-actin was used as an internal control.

### 2.8 Toxicity Tests of UTMD and NO + UTMD

The rat blood plasma samples were collected to analyze the liver function by alanine aminotransferase (ALT), aspartate aminotransferase (AST), and alkaline phosphatase (ALP) and the kidney function by urea and creatinine (Cr). The blood samples (500 µL) were collected from the tail veins. All blood samples were analyzed with a biochemical analyzer (Beckman Coulter Inc., Brea, CA, United States). For histopathology, the heart, liver, spleen, lungs, and kidneys from all groups were fixed in 4% neutral buffered formalin and then embedded in paraffin. Paraffin-embedded tissues were cut into 5-µm-thick sections and stained with H&E to be subsequently examined under a light microscope.

### 2.9 Statistical Analysis

The data are presented as the mean ± standard deviation (SD) and analyzed by GraphPad Prism 8. An unpaired Student's t-test was performed to determine significant differences between the two groups. Multiple comparisons were analyzed by one-way ANOVA. A value of *p<* 0.05 was considered significant.

## 3 Results

### 3.1 Characterization of MBs and NO-MBs and NO Release Under Ultrasound *In Vitro*

**FIGURE 2:**
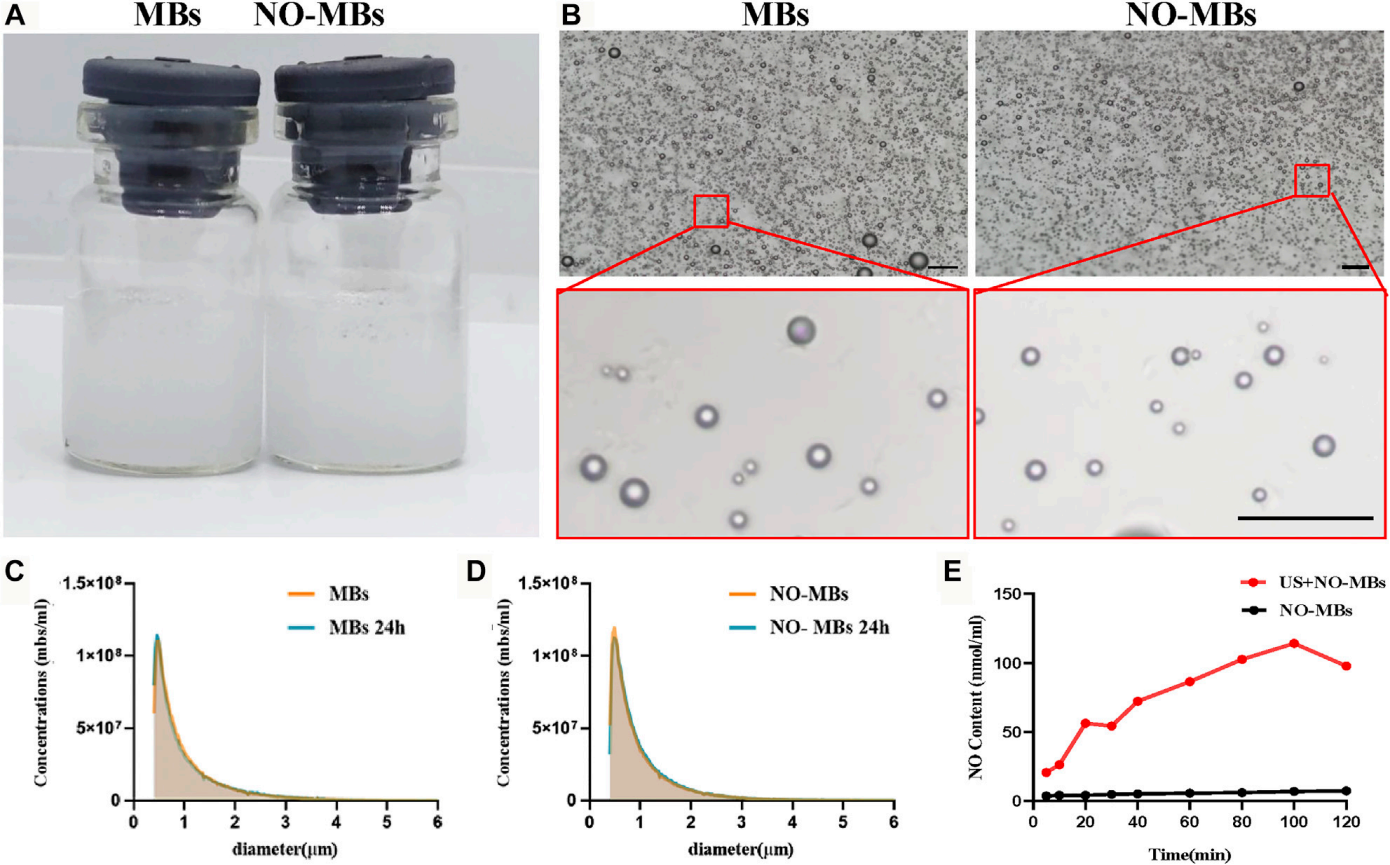
Characterization of MBs and NO-MBs. **(A)** Macroscopic appearance of the prepared MBs and NO-MBs. **(B)** MBs and NO-MBs under optical microscope (scale bar = 10 µm). **(C,D)** The mean diameter and concentration of MBs and NO-MBs. For stability assessment, the average size distribution and concentration of MBs and NO-MBs were measured again after 24 h. **(E)** The release profiles of NO from NO-MBs with US *in vitro*.

Since the small molecular mass of NO results in poor stability of the NO-MBs, we introduced sulfur hexafluoride (SF_6_), a large-molecule gas, to enhance its stability. The MB and NO-MB solutions appeared as uniform curd-like suspensions without differences (Figure 2A). Typically, MBs and NO-MBs show polydisperse spherical morphology without aggregation under a light microscope (Figure 2B). As shown in Figures 2C,D, there were no significant differences between MBs and NO-MBs with respect to the initial concentration (1.10 × 10^8^ MBs/ml vs. 1.19 × 10^8^ MBs/ml) and size distribution (1.068 ± 0.798 µm vs. 1.012 ± 0.712 µm). For the stability assessment, the concentrations and size distribution did not significantly change after 24 h at 4°C, which indicated that the MBs and NO-MBs were stable (Figures 2C,D).

The *in vitro* release profiles of NO-MBs are shown in Figure 2E. The release profile of NO from NO-MBs without US was measured; there was no obvious release, as it reached a maximum content of 7.273 ± 0.856 nmol/ml. However, the release profile of NO with US was significantly higher than without US and reached a maximum content of 114.1 ± 3.7 nmol/ml. These results indicated that NO release from NO-MBs with US was successful.

### 3.2 Release and Specific Delivery of NO to Hind Limb Muscle by UTMD

**FIGURE 3:**
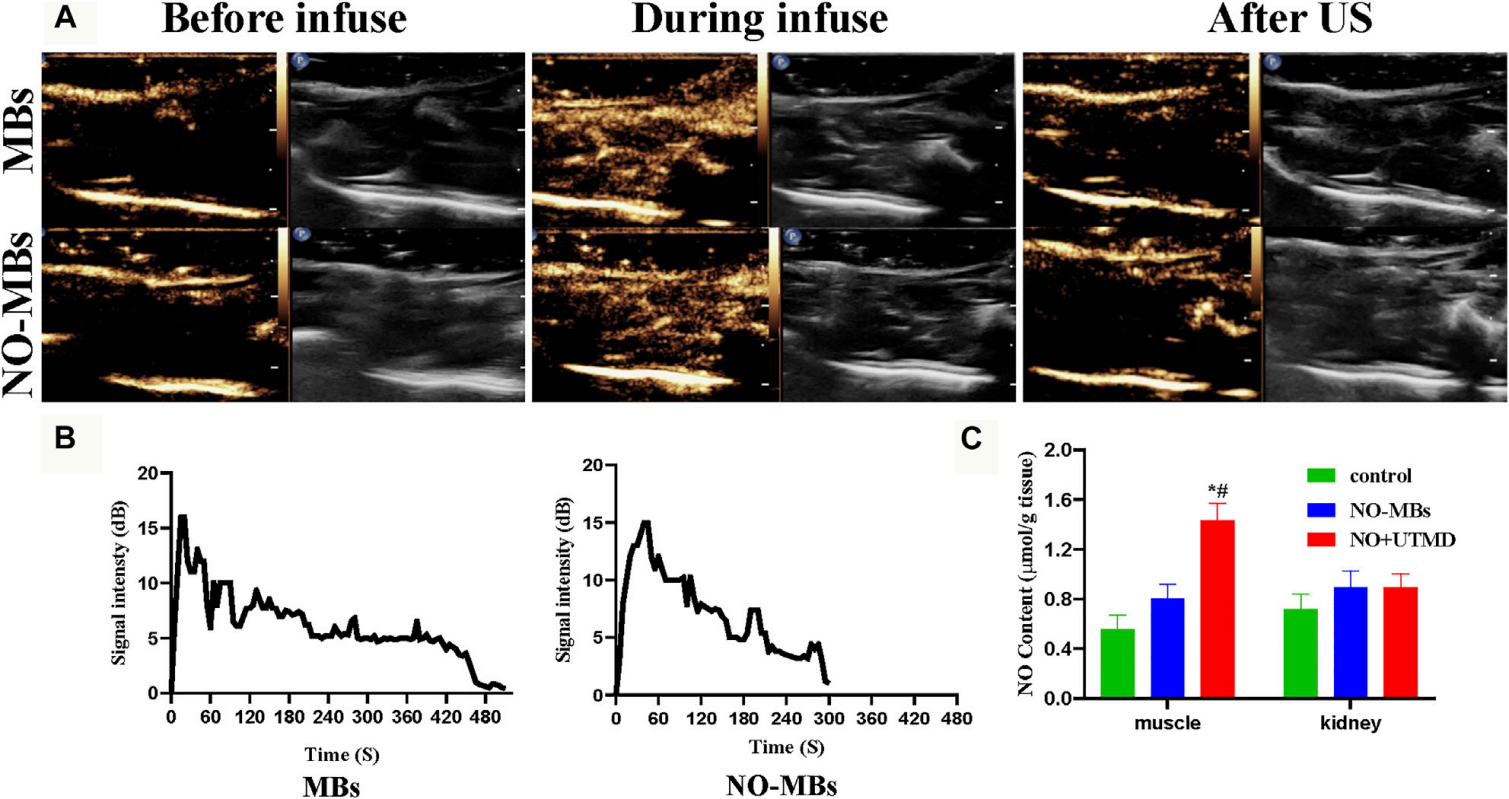
CEUS imaging in rat hind limb. **(A)** CEUS imaging of US-targeted NO-MBs destruction in rat hind limb muscle. **(B)** Signal intensity curves in rat hind limb (without US) of MBs and NO-MBs. **(C)** NO content in different tissues in healthy rats after NO + UTMD treatment.

Figure 3A shows that US successfully destroyed the MBs and NO-MBs in the hind limb muscle. Before infusion, there was no enhanced US signal in the hind limb. A significant increase in the US signal in the hind limb was observed during infusion. After 15 min of US, the US signal was comparable with that before infusion. The *in vivo* circulation times of MBs and NO-MBs were 510 and 300 s, respectively (Figure 3B). The results indicated that MBs and NO-MBs remained stable *in vivo*.

To measure the effects of UTMD in the specific delivery of NO, the skeletal muscle and kidneys were immediately harvested at the end of US radiation, and the results are shown in Figure 3C. The NO content in the hind limb muscle in the NO + UTMD group was higher than in the NO-MB and control groups (1.44 ± 0.13 μmol/g tissue vs. 0.80 ± 0.11 μmol/g tissue vs. 0.55 ± 0.11 μmol/g tissue). By contrast, there was no difference in the NO content in the kidney tissue in the control, NO-MB, and NO + UTMD groups (0.71 ± 0.12 μmol/g tissue vs. 0.89 ± 0.13 μmol/g tissue vs. 0.89 ± 0.11 μmol/g tissue). The results indicated that UTMD can effectively deliver NO to the skeletal muscle.

### 3.3 NO-MBs Decrease Oxidative Stress and Alleviate Apoptosis *In Vitro*

To select a suitable concentration for *in vitro* experiments, the effects of different concentrations of MBs and NO-MBs on cell viability were evaluated by the MTT method. Supplementary Figures S1A,B indicate that MB and NO-MB concentrations of 10^7^ MBs/ml showed some toxic effects on HUVECs and L6 cells, while cells exposed to concentrations of 10^6^ and 10^5^ MBs/ml had a high survival rate, demonstrating the dose-dependent effect of MBs and NO-MBs. In addition, the effect of MBs and NO-MBs was also tested on IRI cells. Supplementary Figures S1C,D show that MB and NO-MB concentrations of 10^6^ and 10^5^ MBs/ml had negligible toxicity on IRI cells. These results indicate that the MBs and NO-MBs had good biocompatibility. Therefore, 10^6^ MBs/ml for MBs and NO-MBs was used for the *in vitro* study.

**FIGURE 4:**
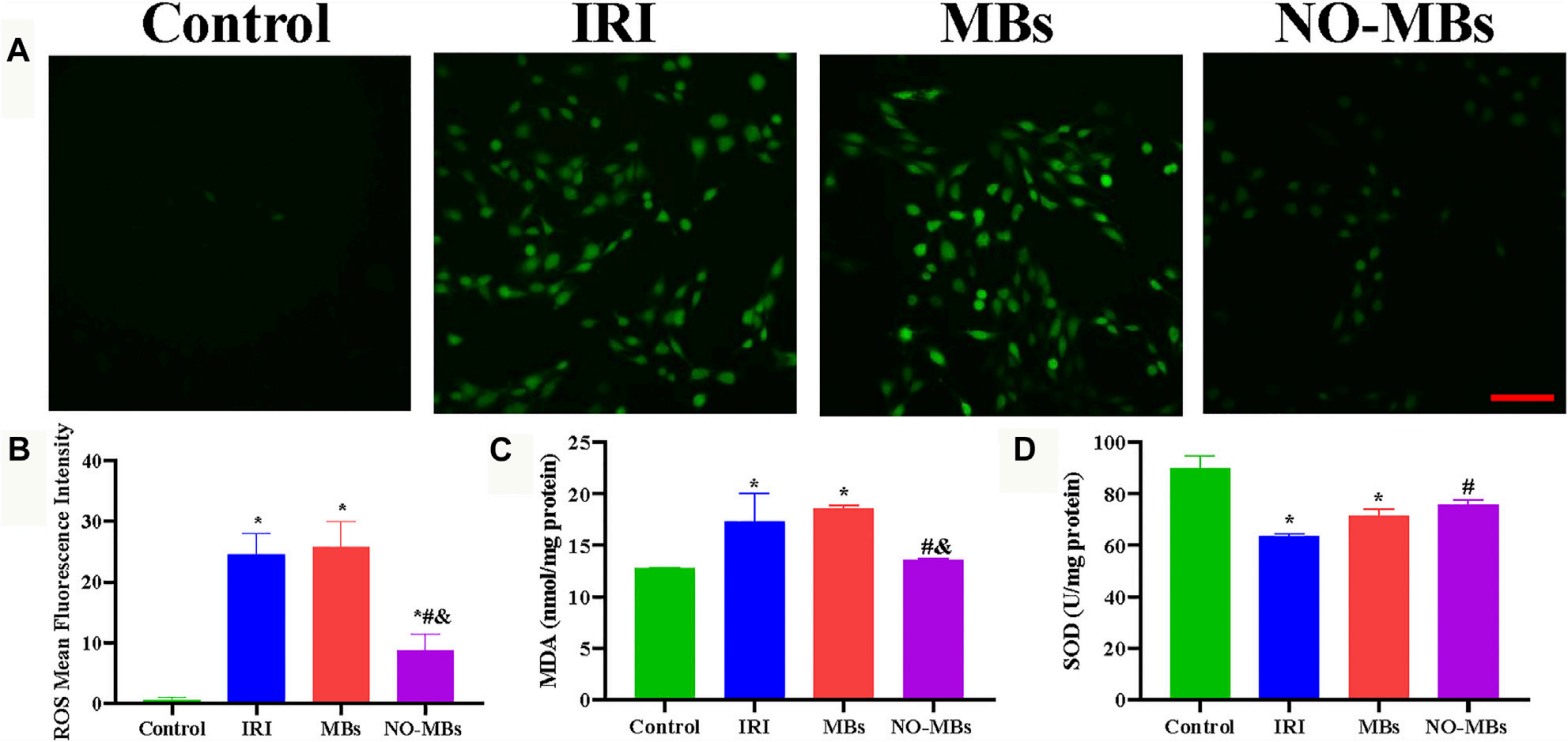
NO-MBs alleviated oxidative stress *in vitro*. **(A)** Representative images of L6 cells stained with ROS (scale bar = 100 µm). **(B)** Quantitation of the mean fluorescence intensity of ROS. **(C)** The level of MDA in the L6 cells. **(D)** The level of SOD in the L6 cells. *p < 0.05 vs. control; #*p* < 0.05 vs. IRI, and *p* < 0.05 vs. MBs, *n* = 3 per group.

To investigate the effect of NO-MBs on hypoxia/reoxygenated L6 cells, biomarkers of oxidative stress, pro-oxidative ROS, and the antioxidants MDA and SOD were analyzed. We found that ROS-positive cells were greatly increased in the IRI group compared to the control group and were comparable with the MBs group. By contrast, ROS-positive cells were substantially decreased after treatment with NO-MBs (Figures 4A,B). IRI increased MDA levels and decreased SOD levels *in vitro* compared to these levels in the control group. No differences were observed in the MDA and SOD contents between the IRI and MB groups. There was an increase in SOD and a reduction in MDA in the NO-MB group compared to the levels in the IRI group (Figures 4C,D). Similar tendencies of these effects were found in cultured HUVECs (Supplementary Figures S2A–D). These findings show that NO-MBs reduced IRI-induced oxidative stress at the cellular level.

**FIGURE 5:**
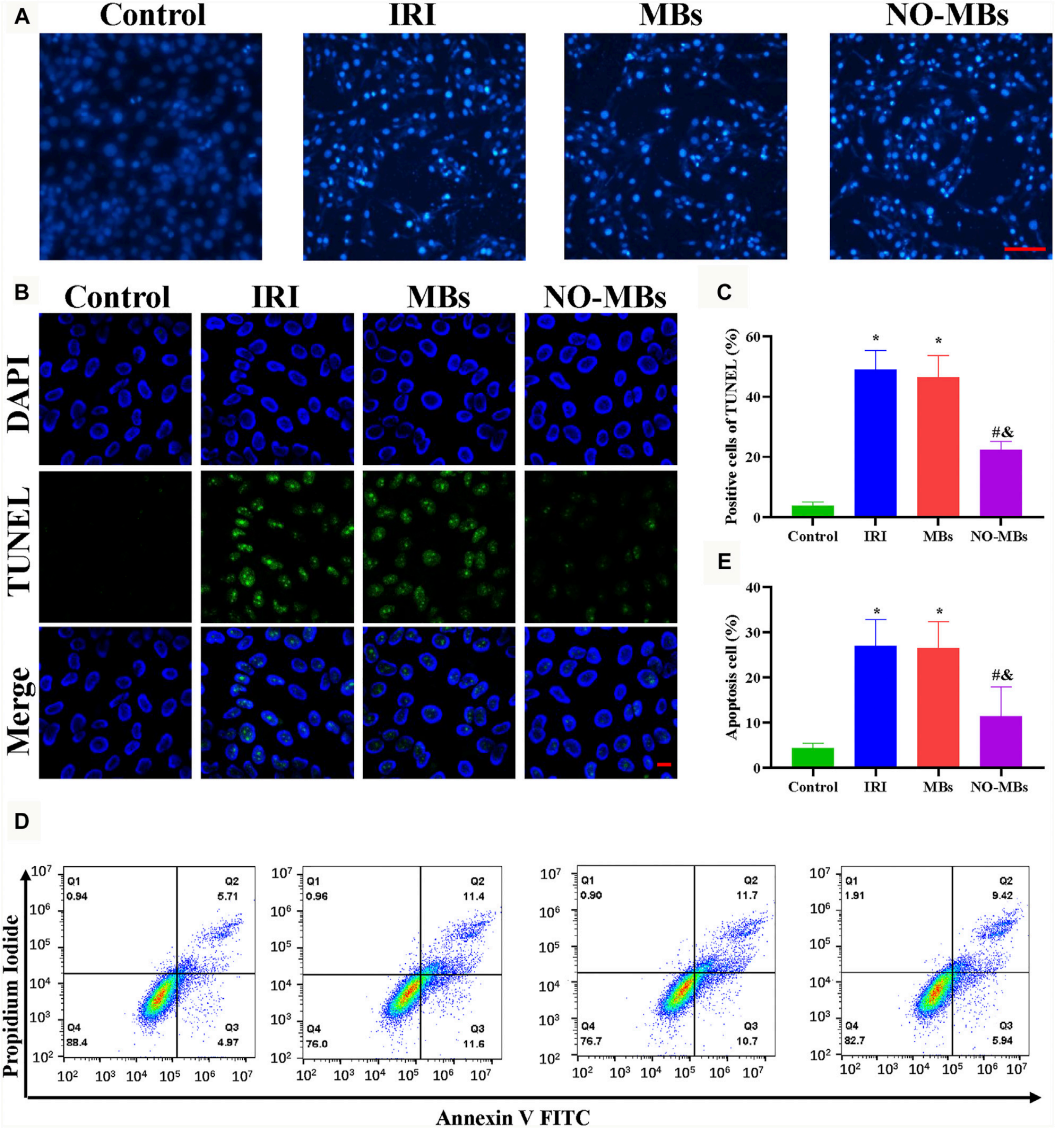
NO-MBs mitigated apoptosis *in vitro*. **(A)** Representative images of L6 cells stained with Hoechst 33258 (scale bar = 100 µm). **(B)** Representative images of L6 cells stained with TUNEL (scale bar = 10 µm). **(C)** Quantitation of apoptotic cells. **(D)** Apoptosis in L6 cells was measured by flow cytometry. **(E)** Quantitative analysis of the flow cytometry. *p < 0.05 vs. control; #*p* < 0.05 vs. IRI, and *p* < 0.05 vs. MB, *n* = 3 per group.

To observe the morphological appearance of IRI-induced apoptotic L6 cells, Hoechst 33258 staining was applied. Morphological features of apoptosis, such as chromatic agglutination and nuclear fragmentation, were observed in the IRI and MB groups compared to the control group. The NO-MB group showed minimal apoptotic changes (Figure 5A). As shown in Figures 5B,C, TUNEL staining was performed to measure the number of apoptotic cells. The number of apoptotic cells increased in the IRI and MB groups compared with the control group. By contrast, there were fewer apoptotic cells in the NO-MB group than in the IRI and MB groups. As shown in Figures 5D,E, cell apoptosis was determined with flow cytometry. We found that cell apoptosis was greatly increased in the IRI group compared to the control group and was comparable to that in the MB group (33.07 ± 7.86% vs. 4.04 ± 0.96% vs. 32.80 ± 9.24%). By contrast, cell apoptosis was significantly decreased after treatment with NO-MBs (11.70 ± 3.93%). Similar tendencies of these effects were found in cultured HUVECs (Supplementary Figures S3A–E). These results suggest that NO-MBs had a protective effect against IRI via an antiapoptotic pathway.

### 3.4 UTMD and NO + UTMD Facilitate Thrombolysis

**FIGURE 6:**
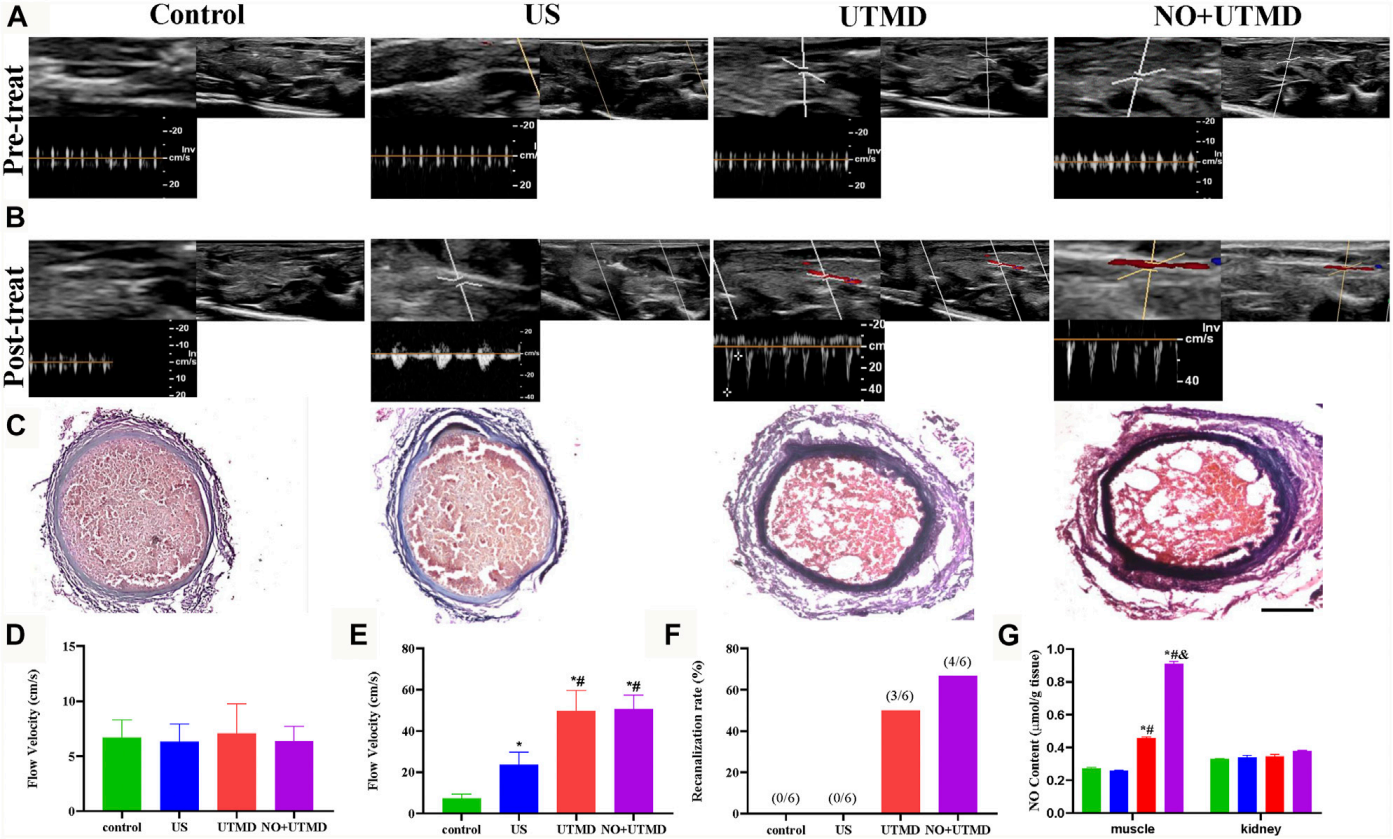
Thrombolytic effect of UTMD and NO + UTMD. **(A)** B-mode and PW Doppler US imaging of rat hind limb before treatment. **(B)** B-mode and PW Doppler US imaging of rat hind limb after treatment. **(C)** Representation pictures of HE staining of the iliac artery (scale bar = 100 µm). **(D)** Quantitative analysis of blood flow velocity before treatment. **(E)** Quantitation of blood flow velocity after treatment. **(F)** Quantitative analysis of recanalization rate after treatment. **(G)** NO content in different tissues in thromboembolism rats after treatment. *p < 0.05 vs. control; #*p* < 0.05 vs. US, and *p* < 0.05 vs. UTMD, *n* = 6 per group.

The effect of UTMD and NO + UTMD on thrombolysis was evaluated by the recanalization rates and blood flow velocities in thromboembolic rats. Large thrombosis in the iliac artery was successfully confirmed by PW US, B-mode US, and H&E staining (Figures 6A,C). Before treatment, the flow velocity was weak and comparable among the four groups, indicating the formation of an occlusive thrombus (Figure 6D). After 15 min of treatment, the flow velocity was comparable in the UTMD (49.65 ± 9.9 cm/s) and NO + UTMD (50.5 ± 6.8 cm/s) groups and was significantly higher in the US (23.7 ± 5.9 cm/s) and control (7.3 ± 2.0 cm/s) groups (Figures 6B,E). Consistently, compared to the recanalization rate of 0% in the US and control groups, UTMD (50.0%) and NO + UTMD (66.6%) showed a great improvement in the recanalization rate (Figure 6F). Iliac artery recanalization was defined as a flow velocity higher than 75% in normal rats. To analyze the effect of UTMD and NO + UTMD on thrombolysis, iliac artery thrombosis structures of all four groups were histologically observed. H&E staining showed clots composed of many erythrocytes and a dense fibrin–platelet meshwork in the control and US groups. The thrombus area in the UTMD and NO + UTMD groups was significantly smaller than in the control and US groups. Notably, there were few microcavities similar in shape to that of an MB (Figure 6C). These results indicate that the effect of NO + UTMD on thrombolysis was as effective as the effect of UTMD on thrombolysis.

### 3.5 Vasorelaxation of the Iliac Artery After UTMD and NO + UTMD

After 15 min of treatment, we measured the internal diameter of the iliac artery. The internal diameter enlarged in the UTMD (0.090 ± 0.008 cm) group compared with the US group (0.065 ± 0.010 cm) and the control group (0.070 ± 0.010 cm) and were comparable to the NO + UTMD (0.095 ± 0.015 cm) group (Supplementary Figures S4A–C).

### 3.6 UTMD Mitigated Oxidative Stress and Apoptosis

**FIGURE 7:**
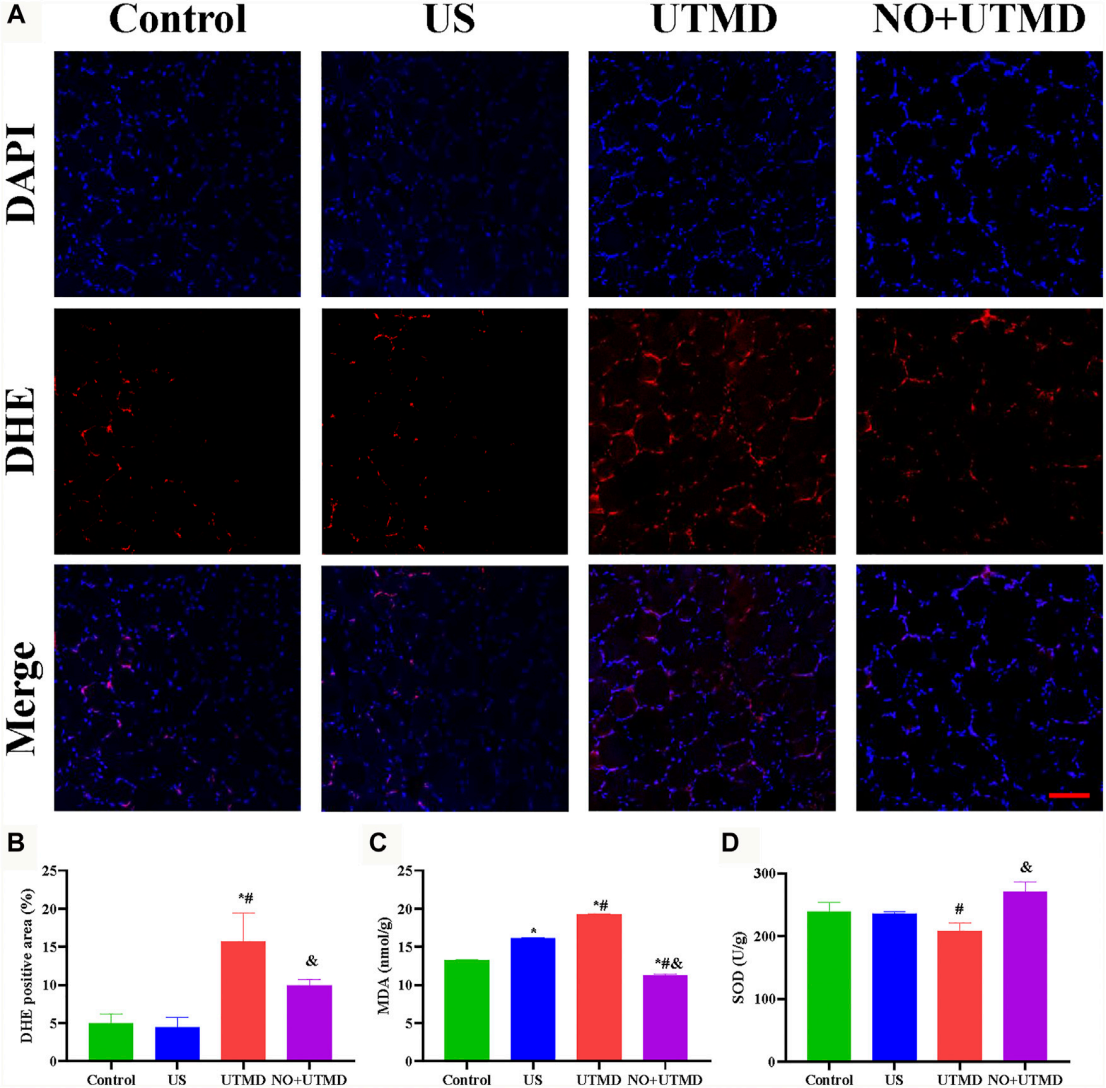
Alleviated oxidative stress in rat hind limb muscle. **(A)** Representative images of DHE staining in hind limb muscle (scale bar = 100 µm). **(B)** Quantitative analysis of the DHE-positive cells in the hind limb muscle. **(C)** The level of MDA in the hind limb muscle after treatment. **(D)** Level of SOD in the hind limb muscle after treatment. *p < 0.05 vs. control; #*p* < 0.05 vs. US, and *p* < 0.05 vs. UTMD, *n* = 6 per group.

**FIGURE 8:**
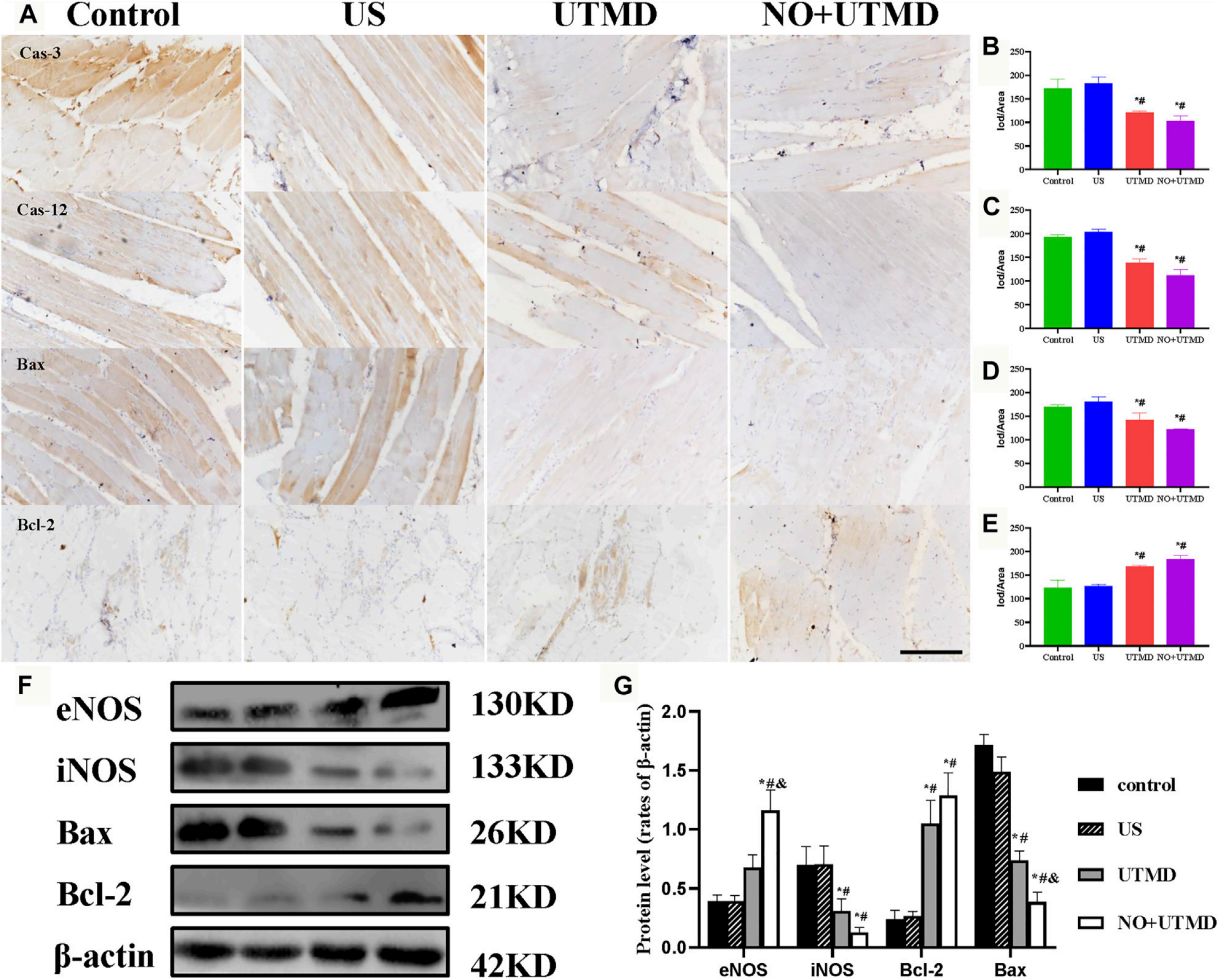
Mitigated apoptosis in rat hind limb muscle. **(A)** Representative images of immunohistochemical staining of caspase-3, caspase-12, Bax, and Bcl-2 expression (scale bar = 100 µm). **(B)** Quantitative analysis of the expression of caspase-3. **(C)** Quantitative analysis of the expression of caspase-12. **(D)** Quantitative analysis of the expression of Bax. **(E)** Quantitative analysis of the expression of Bcl-2. **(F)** Expressions of eNOS, iNOS, Bax, Bcl-2, and *β*-actin in rat hind limb muscle. **(G)** Quantitative analysis of eNOS, iNOS, Bax, Bcl-2, and *β*-actin. *p < 0.05 vs. control; #*p* < 0.05 vs. US, and *p* < 0.05 vs. UTMD, *n* = 3 per group.

To evaluate the effects of NO + UTMD on IRI after thrombolysis, ROS, SOD, and MDA were detected, and the results are shown in Figures 7A–D. The ROS-scavenging ability of the NO + UTMD group was compared to that of the UTMD group without NO as a control for reperfusion. Compared to the control and US groups, reperfusion caused by UTMD led to an increase in ROS and MDA and a decrease in SOD. Moreover, NO + UTMD reduced ROS and MDA and increased SOD in the hind limb muscle, which was in sharp contrast to the effects of UTMD without NO. Thus, NO + UTMD could mitigate IRI after reperfusion. Then, we analyzed the ability of NO + UTMD to protect against IRI and its relevance in decreasing apoptosis. The protein expression of the proapoptotic biomarkers Bax, caspase-3, and caspase-12 was reduced, while that of the antiapoptotic protein Bcl-2 was increased in the UTMD group when compared to the US and control groups. More importantly, there was a significant decrease in caspase-3, caspase-12, and Bax and an increase in Bcl-2 in the NO + UTMD versus UTMD groups (Figures 8A–E). Western blot assays confirmed the trend in Bax and Bcl-2 protein expressions (Figures 8F,G).

### 3.7 UTMD Activated eNOS and NO Generation

The expression of eNOS and iNOS is shown in Figure 8F. Quantitative analyses of eNOS and iNOS are shown in Figure 8G. Greater expression of eNOS was observed in the UTMD group than in the control and US groups. Notably, NO + UTMD exhibited a more powerful ability to stimulate eNOS generation than UTMD. Compared to the control and US groups, iNOS expression was decreased in the UTMD group, and a lower iNOS expression was observed in the NO + UTMD group. As indicated in Figure 6G, the UTMD group had a substantially increased NO content in the muscle when compared to the US group and control group. The NO + UTMD group had an increased NO content in the muscle when compared to the UTMD group. However, the NO content in the kidneys was comparable in the NO + UTMD, UTMD, US, and control groups.

### 3.8 UTMD and NO + UTMD Have Good Biocompatibility

**FIGURE 9:**
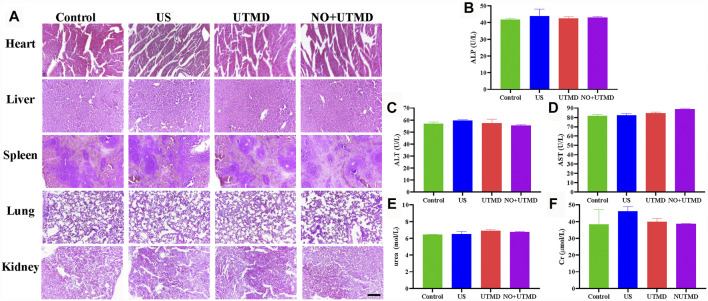
UTMD and NO + UTMD have good biocompatibility. **(A)** Representative images of HE staining of the heart, liver, spleen, lungs, and kidneys after treatment (scale bar = 100 µm). **(B)** Serum biochemical indexes of ALP. **(C)** Serum biochemical indexes of ALT. **(D)** Serum biochemical indexes of AST. **(E)** Serum biochemical indexes of urea. **(F)** Serum biochemical indexes of Cr.

The blood pressure and heart rate were monitored after treatment to appraise the safety of NO + UTMD. Table 1 shows that the blood pressure and heart rate remained constant after treatment. Histology of the heart, liver, spleen, lungs, and kidneys was performed, and the results indicated that UTMD and NO + UTMD did not exert adverse effects on these organs (Figure 9A). The results of the liver and kidney function analyses were within the normal range in the UTMD and NO + UTMD groups (Figures 9B–F).

**TABLE 1:** Blood pressure and heart rate did not change after treatment.

	0 min	5 min	10 min	15 min	20 min	40 min	60 min
Blood pressure (mm Hg)							
Control	120/80	110/75	119/71	118/74	119/72	112/74	125/65
US	118/67	114/78	108/73	111/75	125/85	115/68	117/78
UTMD	123/74	110/80	119/79	109/70	121/83	113/79	125/69
NO + UTMD	117/77	118/74	120/75	123/80	114/75	120/79	126/70
Heart rate (bpm)							
Control	339 ± 14	343 ± 5	345 ± 5	345 ± 10	348 ± 9	344 ± 13	348 ± 7
US	345 ± 14	342 ± 12	344 ± 13	348 ± 9	338 ± 9	347 ± 3	345 ± 13
UTMD	353 ± 7	341 ± 6	344 ± 14	345 ± 9	356 ± 6	347 ± 10	345 ± 9
NO + UTMD	353 ± 7	350 ± 4	346 ± 1	349 ± 7	356 ± 4	344 ± 3	348 ± 4

## 4 Discussion

In the experiment, we showed that NO-MBs with proper proportions of NO and SF_6_ were visible and effectively released therapeutic gas under US. NO-MBs and US were applied to deliver NO into the embolized vessel in the treatment of thrombus and IRI. NO + UTMD had an effect on thrombolysis and mitigated oxidative stress and apoptosis compared to the effects of UTMD. This protective effect was associated with activating eNOS and NO delivery.

Although it may be challenging, the preparation of stable and effective NO-MBs is the keystone in the treatment of IRI. The effect of NO, a gaseous signaling molecule, in IRI has been explored for the past decade in both animal experimental and clinical studies (Bice et al., 2016). However, different levels and locations of NO evoked variable and sometimes opposing effects. At nanomolar or picomolar concentrations, NO may accelerate the progression of tumors by angiogenesis, whereas at micromolar concentrations, NO may be a useful chemotherapeutic agent by inducing oxidative stress (Mocellin et al., 2007). In our study, we found that NO was encapsulated in MBs and released under US at a nanomolar concentration. We hypothesized that the biological effects of NO depend on its circulation time. The circulation time of NO was significantly improved when encapsulated in MBs. The release of NO under US from NO-MBs is fast, and half of the amount is released in 20 min. Such a release profile is quite satisfactory in the treatment of immediate IRI after reperfusion. In healthy rats, we demonstrated that NO-MBs were visually tracked in the hind limb muscle and destroyed under US by CEUS. Importantly, we found that the NO content was increased after NO + UTMD treatment, indicating that the bioavailability of NO for targeted tissue was enhanced. Although inhaled NO or administration of an NO donor potently decreased IRI and apoptosis, NO + UTMD may have greater efficacy due to enhanced bioavailability (Liu et al., 2007). There are two protective mechanisms by which NO + UTMD can increase the bioavailability of NO to damaged tissues. One possible mechanism is that the MB shell protects NO from endogenous scavengers. Alternatively, according to the study by Postema et al., a region without red blood cells (RBCs) located close to the endothelium could protect NO from being consumed by RBCs (Liao et al., 1999; Postema et al., 2006). The bioavailability of NO may be enhanced by employing acoustic radiation force to push the NO-MBs to the no-RBC region.

The data from the assessment of the increased recanalization rate and flow velocity and H&E staining of the iliac artery indicated that UTMD and NO + UTMD had a substantial thrombolysis effect. Current research has shown that the mechanisms of US enhancement of thrombolysis are complicated, with a particular focus on acoustic cavitation (de Saint Victor et al., 2014; Postema et al., 2006). The acoustic cavitation effect can be divided into stable cavitation and inertial cavitation. When experiencing stable cavitation, the MBs oscillate in a regular and continuous manner and transfer momentum to the surrounding fluid, resulting in localized eddying motion (de Saint Victor et al., 2014). This microflow effect may cause damage to the thrombus surface. When undergoing inertial cavitation, MBs collapse and create high-speed liquid jets, causing the fibrin network to break (Weiss et al., 2013). Microcavities of different sizes can be seen in the histopathological examination of the iliac artery, which contributes to acoustic cavitation. The microcavities indicated that acoustic cavitation is effective in thrombolysis. The effect of acoustic cavitation on thrombolysis largely depends on the US parameters and MBs. MB properties, including the gas encapsulated within the bubble, shell material, and mean diameter, have been shown to be important during thrombolysis. NO is bioactive and unstable when dissolved in the blood; therefore, a firm shell and large-molecule gas were introduced to stabilize NO-MBs, which is feasible in theory (Kim et al., 2014; Sutton et al., 2014). After 24 h of storage, we found no differences in the size distribution and concentration of NO-MBs and MBs, indicating good stability of NO-MBs. The only difference between MBs and NO-MBs was the gas contained in the bubbles. Thus, it is reasonable to conclude that the thrombolytic efficiency was comparable between MBs and NO-MBs despite their different gas cores.

Accumulated evidence has shown that acoustic cavitation can significantly promote thrombolysis, but the effect on shear–wave-sensitive vascular endothelium remains to be explored. In rabbit hind limb acute ischemia (Atar et al., 2007) or coronary occlusion in pigs (Siegel et al., 2004), low-frequency US (27 kHz) without MBs can effectively improve blood perfusion through the production of NO. Similarly, therapeutic US (1 MHz) rescues ischemia-induced angiogenesis via the eNOS signaling pathway (Huang et al., 2014; Lu et al., 2016a; Lu et al., 2016b). In contrast to these studies, the blood flow in the hind limb was improved in the US group; however, the expression of eNOS was not increased. The duration of US irradiation may be the main reason. With the addition of MBs, the NO content and blood flow were increased in the UTMD group. Our data indicated that these effects may most likely be mediated by acoustic cavitation and activation of eNOS, consistent with previous studies (Belcik et al., 2015). Moreover, it was found that inhibition of eNOS decreased blood perfusion caused by US combined with MBs (Belcik et al., 2015; Belcik et al., 2017). To date, no study has investigated the effect of exogenous NO during acoustic cavitation. Our work is unique in that we specifically applied the model of a completely occluded thrombus, where occluded thrombus was created rather than microthrombi. Our study is also distinct in that we added exogenous NO to the MBs and uncovered the effects of NO in mitigating IRI.

The occurrence of IRI after rapid reperfusion is associated with the generation of ROS and cell apoptosis (Duehrkop and Rieben, 2014). We found that apoptosis and oxidative stress were mitigated in hypoxia/reoxygenated cells in the NO-MB group, suggesting the potential of NO in mitigating apoptosis and oxidative stress *in vivo*, which had been proven in previous studies (Wollert and Drexler, 2002; Razavi et al., 2005; Liu et al., 2007). To investigate the potential of NO-MBs in the treatment of oxidative stress and apoptosis *in vivo*, US was used to specifically deliver NO-MBs to the damaged tissues. It is well-known that stimulated endothelium can release NO into vessels by activating eNOS, but its concentration is too small and half-life too short to prevent IRI. Therefore, it may be feasible to activate eNOS and provide supplemental NO. Our data showed that oxidative stress and apoptosis were mitigated in the hind limbs treated with NO + UTMD compared to the UTMD group, which may have contributed to the activation of eNOS and increased the bioavailability of NO. The overexpression of the eNOS protein increased the concentration of NO in the UTMD group, particularly in the NO + UTMD group. These observations indicated that NO + UTMD probably mitigated IRI through the activation of eNOS and increased the bioavailability of NO. Conventional NO donors may cause undesired side effects such as reduced blood pressure and increased heart rate. The delivery system for NO that we designed here is specific to damaged tissues and does not influence blood pressure or heart rate, which may prevent systemic side effects. The dominant conclusion from these data was that NO can mitigate IRI and play a role in UTMD.

There are several limitations to our experiment. First, the number of samples used in our study is less. Second, we alleviated IRI using NO-MB administration with fixed US parameters and time, and these influencing factors on IRI need to be investigated further. Finally, this study only evaluated the therapeutic effect of NO + UTMD within 24 h, and the systemic effects and long-term effects need to be further analyzed.

## 5 Conclusion

In summary, UTMD is effective in enhancing thrombolysis while causing eNOS activation and NO release. NO-MBs achieved large vessel thrombolysis and protection against IRI. Our study provides a superior option for the clinical translation of NO therapeutic applications.

## Data Availability

The original contributions presented in the study are included in the article/Supplementary Material; further inquiries can be directed to the corresponding authors.
