# Supplementary material for: Ultrasound-Induced Destruction of Nitric Oxide–Loaded Microbubbles in the Treatment of Thrombus and Ischemia–Reperfusion Injury
Source: Front Pharmacol. 2022 Jan 4;12:745693. doi: 10.3389/fphar.2021.745693 (PMC8785684; doi:10.3389/fphar.2021.745693)

Supplementary Material


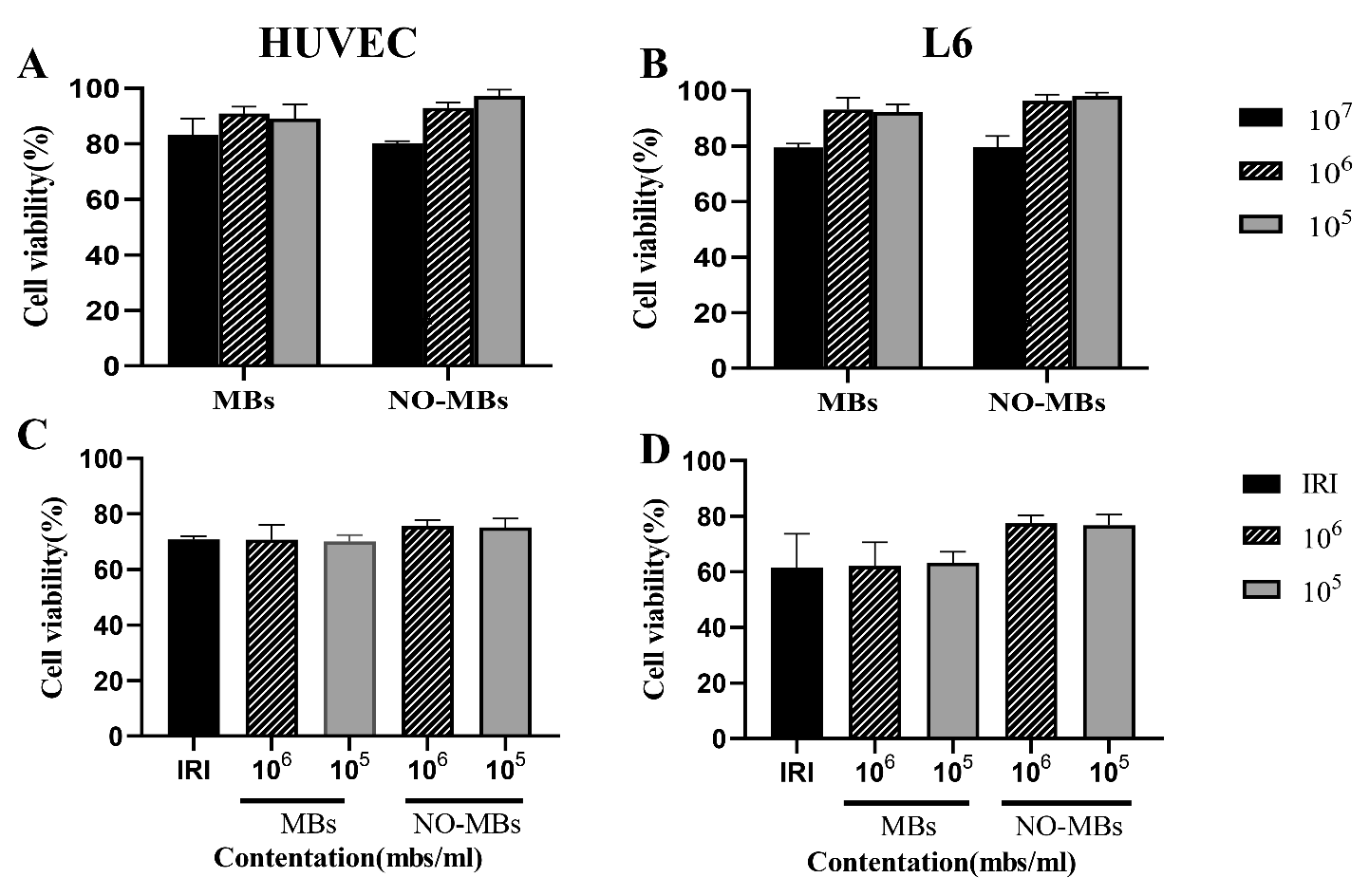


**Supplementary Figure 1. Effect of MBs and NO-MBs on cell viability** (A) Mtt assay showed cell viability of HUVECs after 12h treatment with MBs and NO-MBs. (B) Mtt assay showed cell viability of L6 cells after 12h treatment with MBs and NO-MBs. (C) Mtt assay showed cell viability of hypoxia-predisposed HUVECs after 12h treatment with MBs and NO-MBs. (D) Mtt assay showed cell viability of hypoxia-predisposed L6 cells after 12h treatment with MBs and NO-MBs.

**Supplementary Figure 2** NO-MBs alleviated oxidative stress *in vitro*. (A) Representative images of HUVECs stained with ROS (scale bar= 100µm). (B) Quantitation of the mean fluorescence intensity of ROS. (C) The level of MDA in the HUVECs. (D) The level of SOD in the HUVECs. **P*<0.05 vs. control; #*P*<0.05 vs. IRI, &*P*<0.05 vs. MBs, n=3 per group.


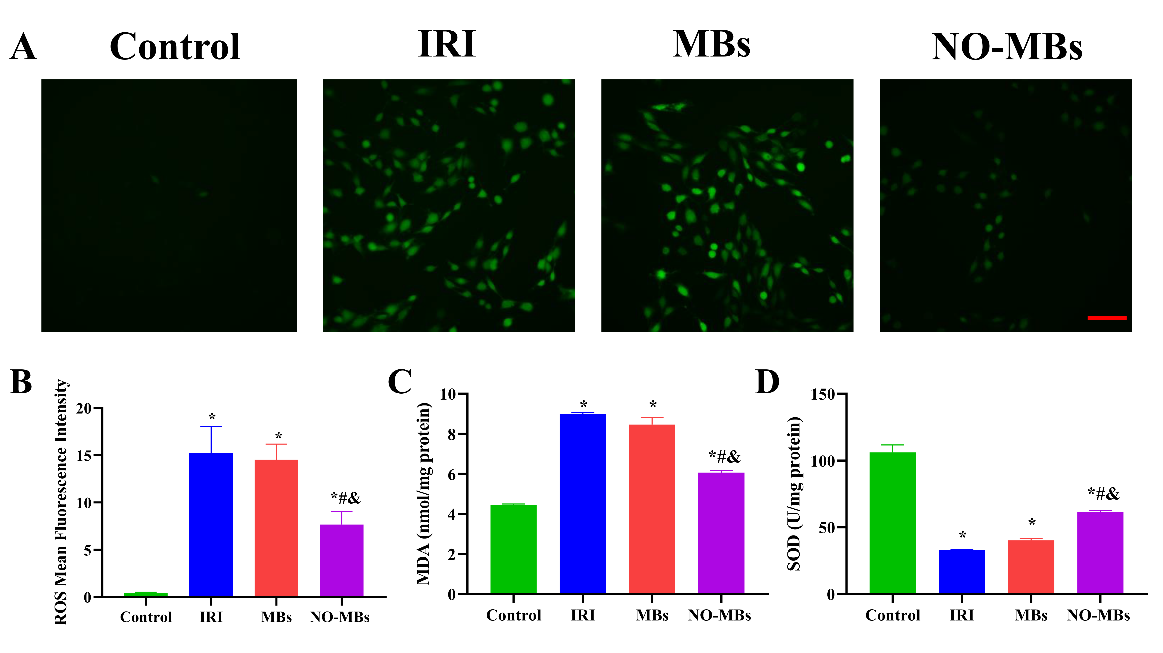


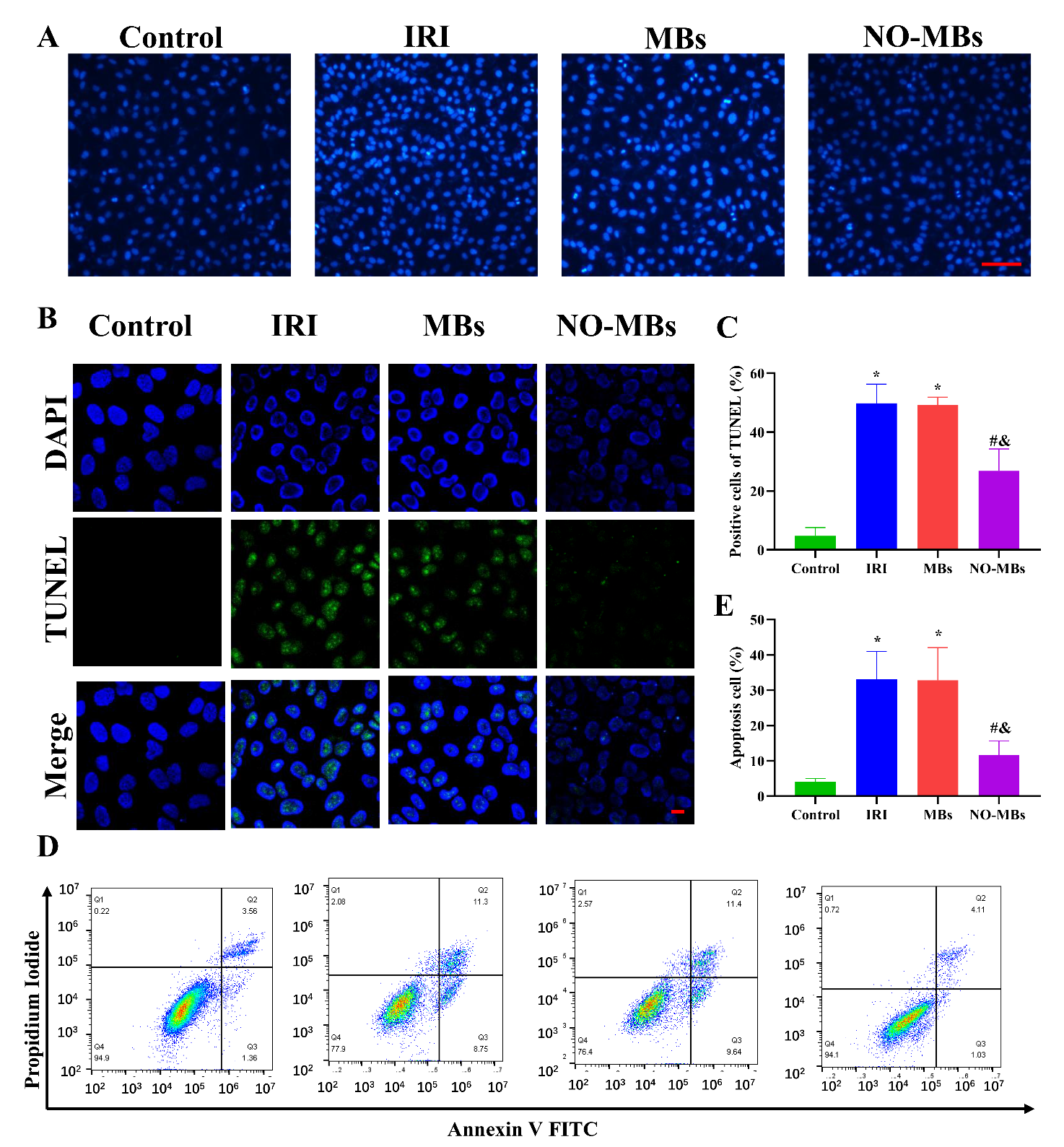


**Supplementary Figure 3.** NO-MBs mitigated apoptosis *in vitro*. (A) Representative images of HUVECs stained with Hoechst 33258 (scale bar= 100µm). (B) Representative images of HUVECs stained with TUNEL (scale bar= 10µm). (C) Quantitation of the apoptotic cells. (D) The apoptosis in HUVECs was measured by flow cytometry. (E) Quantitative analysis of the flow cytometry. **P*<0.05 vs. control; #*P*<0.05 vs. IRI, &*P*<0.05 vs. MB, n=3 per group.

**Supplementary Figure 4. iliac artery vasorelaxation as a treatment.** (A) Ultrasound imaging of rat iliac artery. (B) Measurement of internal diameter of iliac artery. (C) Quantitation of internal diameter of iliac artery after treatment. **P*<0.05 vs. control; #*P*<0.05 vs. US, &*P*<0.05 vs. UTMD, n=6 per group.


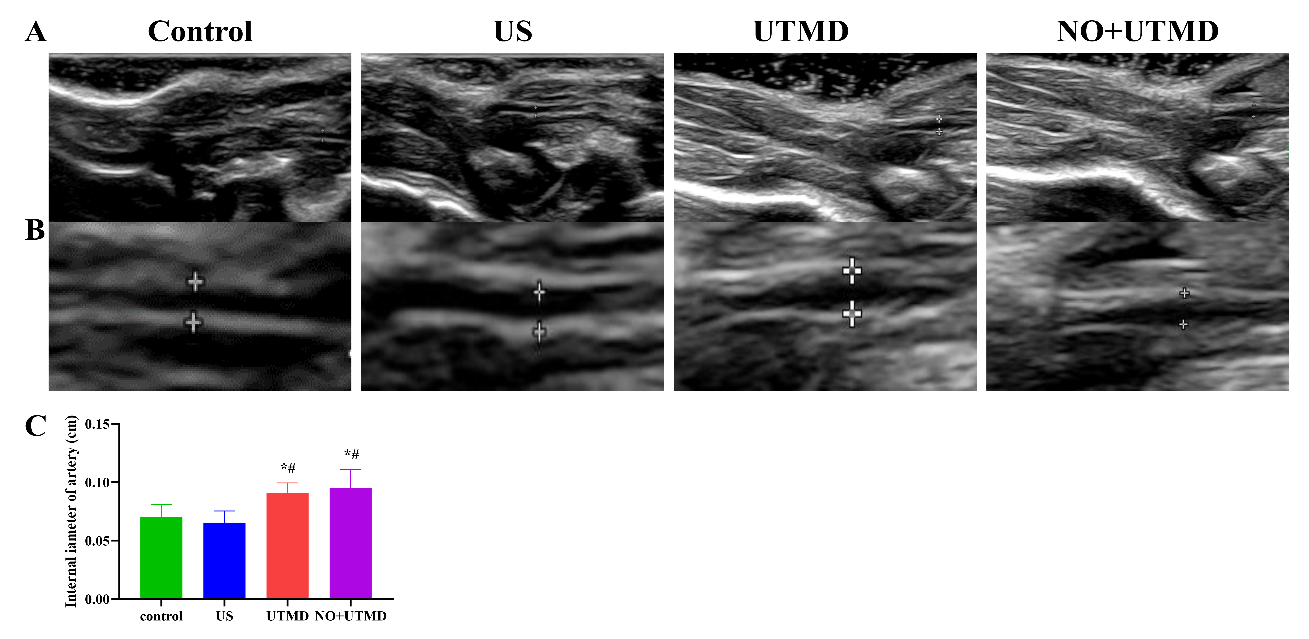

Supplement: Supplementary file 1 [file DataSheet1.docx]
